# Pharmacological analysis of transmission activation of two aphid-vectored plant viruses, turnip mosaic virus and cauliflower mosaic virus

**DOI:** 10.1038/s41598-019-45904-7

**Published:** 2019-06-28

**Authors:** Edwige Berthelot, Jean-Luc Macia, Alexandre Martinière, Alexandre Morisset, Romain Gallet, Stéphane Blanc, Mounia Khelifa, Martin Drucker

**Affiliations:** 1 BGPI, INRA Centre Occitanie, SupAgro, CIRAD, Université de Montpellier, Campus International de Baillarguet, 34398, Montpellier, Cedex 5 France; 2 Semences Innovation Protection Recherche et Environnement, rue des Champs Potez, 62217 Achicourt, France; 3 Fédération Nationale des Producteurs de Plants de Pommes de Terre, 43-45 rue de Naples, 75008 Paris, France; 4 Present Address: BPMP, CNRS, INRA, SupAgro, Université de Montpellier, 2 place Viala, 34060, Montpellier, Cedex 2 France; 5 SVQV, INRA Centre Grand Est, Université de Strasbourg, 28 rue de Herrlisheim, 68000 Colmar, France

**Keywords:** Viral transmission, Biotic

## Abstract

Turnip mosaic virus (TuMV, family *Potyviridae*) and cauliflower mosaic virus (CaMV, family *Caulimoviridae*) are transmitted by aphid vectors. They are the only viruses shown so far to undergo transmission activation (TA) immediately preceding plant-to-plant propagation. TA is a recently described phenomenon where viruses respond to the presence of vectors on the host by rapidly and transiently forming transmissible complexes that are efficiently acquired and transmitted. Very little is known about the mechanisms of TA and on whether such mechanisms are alike or distinct in different viral species. We use here a pharmacological approach to initiate the comparison of TA of TuMV and CaMV. Our results show that both viruses rely on calcium signaling and reactive oxygen species (ROS) for TA. However, whereas application of the thiol-reactive compound N-ethylmaleimide (NEM) inhibited, as previously shown, TuMV transmission it did not alter CaMV transmission. On the other hand, sodium azide, which boosts CaMV transmission, strongly inhibited TuMV transmission. Finally, wounding stress inhibited CaMV transmission and increased TuMV transmission. Taken together, the results suggest that transmission activation of TuMV and CaMV depends on initial calcium and ROS signaling that are generated during the plant’s immediate responses to aphid manifestation. Interestingly, downstream events in TA of each virus appear to diverge, as shown by the differential effects of NEM, azide and wounding on TuMV and CaMV transmission, suggesting that these two viruses have evolved analogous TA mechanisms.

## Introduction

Viruses are obligate intracellular parasites and therefore depend completely on the machinery and metabolism of a host cell to accomplish the different steps of their infection cycle. Among these, the host-to-host transmission is a crucial step allowing maintenance and spread of the virus in the environment. Many viruses of animals and most viruses of plants rely on vectors for host-to-host transmission. The most important vector groups are piercing-sucking arthropods such as blood-feeding mosquitoes and ticks for animal viruses, and sap-feeding hemipteran insects for plant viruses, all acting as “flying syringes” and so suited ideally to uptake, transport and inoculate viruses and other pathogens^1,2^. Aphids are the major vectors for plant viruses and responsible for the dissemination of 70% of all plant virus species^3^.

Plant viruses are usually acquired with the food when vectors feed on plants. Depending on the virus-vector interaction, two transmission modes are distinguished. In the circulative mode, viruses traverse the intestine and cycle through the body of the vector to invade the salivary glands. Then they are inoculated together with saliva into a new host. In the non-circulative mode, viruses do not need to cycle through the vector, but are retained in and released from the vector mouthparts (reviewed in^4^). In the helper strategy of non-circulative transmission, the formation of transmissible complexes is mandatory for transmission. Transmissible complexes are composed of the virus particle and the helper component, a viral non-structural protein mediating binding of the virion to the vector mouthparts (reviewed in^5^).

**Figure 1:**
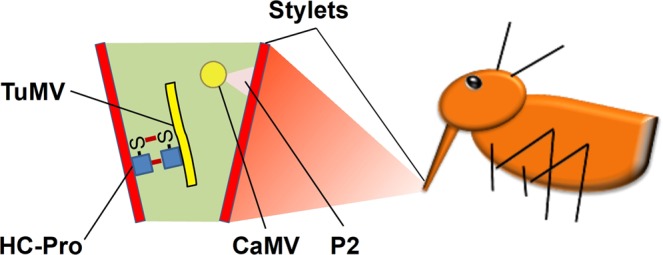
Schemata of the transmissible complexes of TuMV and CaMV in the aphid vector (not drawn to scale). The TuMV transmissible complex is composed of the filamentous TuMV particle and the helper component HC-Pro (left), and the CaMV transmissible complex is composed of the icosahedric CaMV particle and the helper component P2. Both helper components contain a virion binding domain that interacts with the viral capsid and a vector binding domain that interacts with yet unidentified receptors on the cuticle lining the interior of the stylets, the proboscis-like mouthparts of aphids (shown to the right). The aphid is adapted from^7^.

Recent studies on two non-circulative aphid-transmitted viruses using the helper strategy, cauliflower mosaic virus (CaMV, family *Caulimoviridae*) and turnip mosaic virus (TuMV, family *Potyviridae*), showed that vector feeding activity induces formation of transmissible complexes in infected plant cells within seconds^6–8^. This suggests that these two viruses switch transiently from a replication/accumulation mode to a transmission mode, a phenomenon named transmission activation (TA)^9^. The transmissible complexes of TuMV and CaMV are functional, but not biochemical or structural analogues (Fig. 1). The TuMV transmissible complex is composed of the filamentous virus particle and the helper component HC-Pro. HC-Pro is a ~50 kD multifunctional protein involved not only in vector-transmission, but also in suppression of gene silencing and other plant defense reactions, in viral movement and in the processing of the viral precursor polyprotein^10,11^. The CaMV transmissible complex consists of the icosahedric virus particle and the helper component P2, an 18 kD protein whose only function is transmission^12,13^. P2 and TuMV HC-Pro are thus totally different proteins.

For TA to occur, a virus must somehow ‘sense’ the presence of the aphid and then ‘respond’ to aphid feeding activity by forming transmissible complexes. This is only possible via (ab)using the plant sensory system and associated signaling cascades. Since aphid activity on plants induces plant defense responses (reviewed in^14^), it is likely that TuMV and CaMV deviate plant defense reactions to trigger the formation of transmissible complexes. Further, because TA happens within seconds, viruses might interfere with early signaling steps. The earliest event in establishment of plant defenses is recognition by the plant of various stresses. This is achieved in general by specific plant pathogen recognition receptors (PRR) that recognize conserved pathogen associated molecular patterns (PAMP). Though this overall scheme applies likely also to plant-aphid interactions, it should be noted that both PRR and PAMP are yet unidentified in this specific case. Transduction of the recognition signal is then mediated by rapid calcium signaling^15^. Recently, it has been shown that aphid punctures elicit calcium waves that might play a role in signaling^16^. Reactive oxygen species (ROS) also play a significant role in early plant defense reactions and calcium and ROS signals most likely interweave^17,18^. The initial calcium and/or ROS fluctuations trigger downstream reactions that ultimately establish PAMP-triggered immunity (PTI)^19^.

Since calcium and ROS elevations are the earliest plant responses to pathogen presence, we hypothesized that plant viruses might deviate them for TA. Consistently, we showed recently that transmission of TuMV is inhibited by the calcium channel blocker LaCl_3_ and activated by the ROS H_2_O_2_ ^8^. Whether TA of CaMV is also dependent on calcium and ROS or on other pathways is unknown. Therefore, we used here a pharmacological approach to compare TA of these viruses. Our results suggest that TA of the two viruses have initial steps in common, but then rely on different downstream mechanisms.

## Result and Discussion

**Figure 2:**
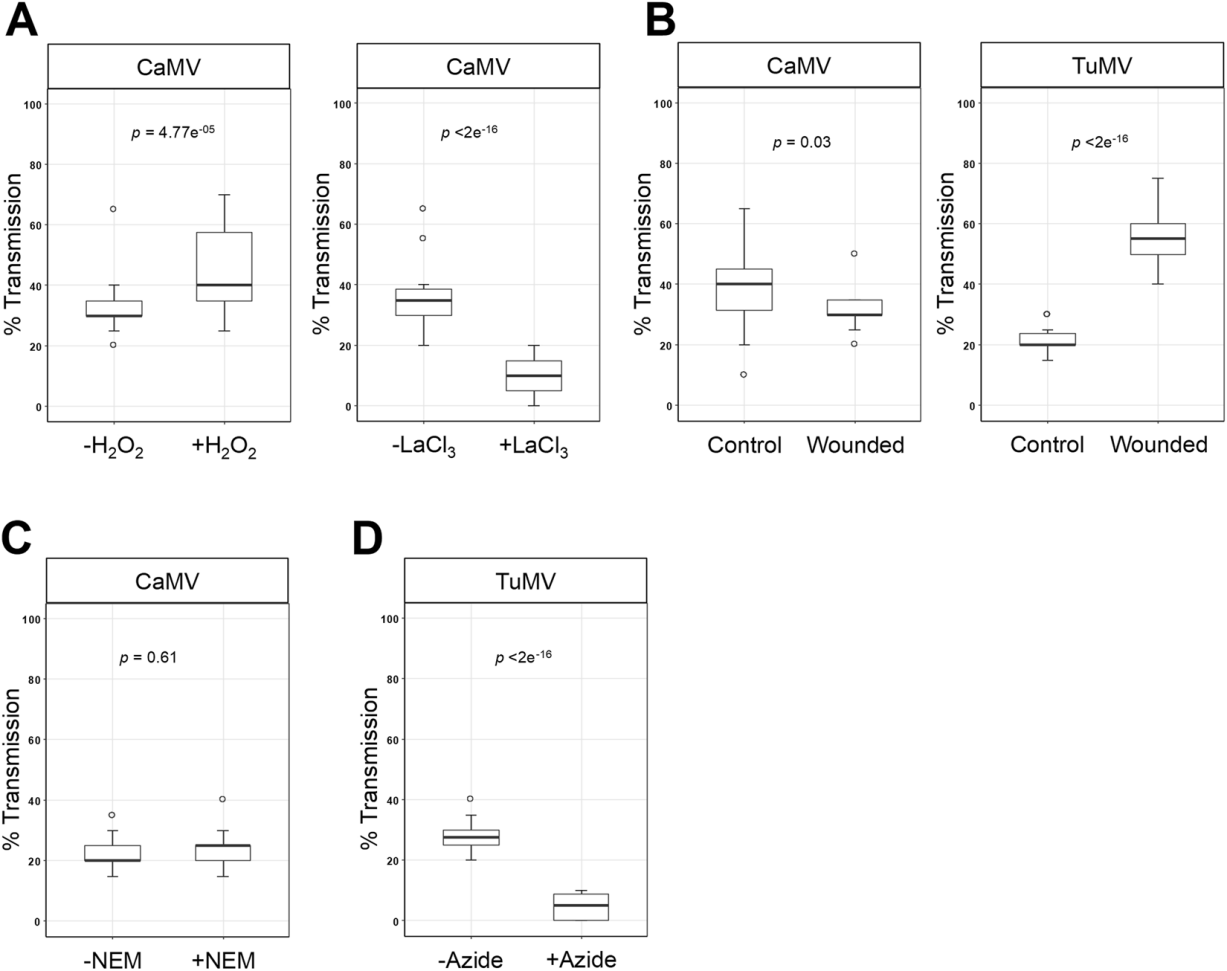
Effect of different treatments on aphid transmission of CaMV and TuMV. (**A,B**) Effect of the ROS H_2_O_2_ and the calcium channel blocker LaCl_3_ on plant-to-plant transmission of CaMV (**A**). Turnip leaves were sprayed with 10 mM H_2_O_2_ or LaCl_3_ solutions or water and incubated for 30 min before transmission tests. (**B**) Effect of wounding on plant-to-plant transmission of TuMV and CaMV. Turnip leaves were wounded by inflicting cuts with a razor blade and immediately used for transmission. (**C**) Effect of the thiol reducing agent NEM on protoplast-to-plant transmission of CaMV. Protoplasts were incubated with 3 mM NEM for 20 min before the transmission tests. (**D**) Effect of azide on protoplast-to-plant transmission of TuMV. Protoplasts were incubated with 0.02% azide for 40 min and then employed in transmission assays. For all transmission tests, means of infected test plants are calculated from a pool of three independent experiments (see Materials and Methods). *p* designates p-values obtained in generalized linear models. Each graph shows medians and quartiles. The whiskers represent sample ranges. The open circles show the outlier samples. We verified that NEM and azide treatment of protoplasts did not change protoplast viability (Supplementary Source file 2). The raw data of the transmission tests are presented in Supplementary Source file 3.

H_2_O_2_ and calcium that activate TuMV transmission are general signaling molecules. Thus, they might be involved in TA of CaMV as well. Therefore, we tested the effect of H_2_O_2_ and the calcium channel blocker LaCl_3_ on plant-to-plant transmission of CaMV. Just as for TuMV, H_2_O_2_ increased and LaCl_3_ decreased transmission of CaMV significantly (Fig. 2A).

We have shown previously that wounding stress induces the typical TA response of CaMV, i.e. formation of P2 networks in infected cells^7^. However, we did not assess transmission in the previous experiment. Therefore, we repeated the experiment to record CaMV transmission and extended the experiment to TuMV transmission. For this, TuMV and CaMV infected leaves on intact plants were wounded by superficial scratching with a razor blade and then employed in aphid transmission tests. Wounding stress increased transmission of TuMV significantly and decreased transmission of CaMV significantly (Fig. 2B). The results indicate that stress response pathways might be involved in TA but affect transmission of TuMV and CaMV differently.

We showed in previous work that N-ethylmaleimide (NEM) inhibited transmission of TuMV^8^ and that sodium azide activated transmission of CaMV^7^ but the effect of these substances on the transmission of the respective other virus was not tested. Consequently, we tested the effect of NEM on CaMV transmission and that of azide on TuMV transmission. Because of the toxicity of the substances and to minimize contact of aphids with them, we used protoplasts prepared from infected plants as virus source. This system has previously been used successfully to characterize aphid transmission of CaMV and TuMV^7,8,20^. Unlike TuMV transmission, NEM did not change CaMV transmission significantly (Fig. 2C). This suggests that formation of intermolecular or intramolecular cysteine bridges that are essential for TuMV transmission^8^ are not important for CaMV transmission. Indeed, TuMV TA depends on cysteine-mediated oligomerization of the helper component HC-Pro that is thought to be the HC-Pro conformation able to interact with TuMV particles for formation of transmissible complexes^8^. The CaMV helper component P2, on the other hand, does not contain any cysteine residues, ruling out a similar mechanism. However, CaMV virion-associated P3 and the capsid protein contain cysteines, and the conformation of P3 has been suggested earlier to be controlled by an intermolecular cysteine ring^21^. Although it cannot be ruled out, our results do not indicate a role of this in CaMV transmission.

Azide treatment that boosts CaMV transmission^7^ had the opposite effect on TuMV transmission, which it inhibited strongly (Fig. 2D). The mode of action of azide on transmission is difficult to assess since it has pleiotropic effects on cells. It inhibits v-type ATPases^22^, catalases, peroxidases and cytochrome oxidase, and thereby the generation of ATP and thus depletes cells of energy (reviewed in^23^). It can also complex with and inhibit other heavy metal containing enzymes, react with amines and many other diverse effects of azide are reported in the literature^24–26^. Therefore, we do not propose a mechanistic explanation of the effect of azide on TA of the two viruses. However, due to its opposing effects on TuMV and CaMV transmission, we conclude that this compound affects different steps in TA of the two viruses.

Taken together, our analysis presents evidence that calcium and ROS are involved in TA of TuMV and CaMV. This indicates that TA might be triggered by the early steps of PAMP-triggered immunity, when plant PRR receptors recognize PAMP and induce calcium signaling and ROS production that itself can also act as a signal. It is reasonable to assume that yet unidentified aphid-associated molecular patterns are recognized by likewise unknown PRR and induce calcium and ROS signals that are somehow ‘eavesdropped’ by TuMV and CaMV. In this context it is worth noting that interrogation of transcriptome databases with Genevestigator^27^ indicated that several calcium-related proteins, cysteine-rich receptor-like protein kinases, and ROS-related proteins are deregulated in TuMV-infected Arabidopsis (see Supplementary Source file 1; there is no data available for CaMV-infected plants). It is tempting but speculative to propose that deregulation of some of these genes might be related to TA. Vincent and coworkers^16^ described calcium waves triggered by aphid punctures and presented evidence that the receptor kinase BAK1, one of the hubs linking various PRR with downstream events^28^, is involved in this process. Interestingly, BAK1 had been identified before as being involved in aphid plant interactions^29^. Thus, there is strong evidence for the existence of a PTI-like aphid-triggered immunity (ATI). While we can speculate that ATI could be used by some viruses to initiate TA, we cannot elaborate further, at this point, on how TuMV and CaMV intercept with it. They could use the same or different PRR (assuming there is more than one aphid elicitor) and downstream pathways, just as the highly conserved bacterial effectors flagellin and elongation factor Tu do^30^. It will be a challenge for the future to dissect ATI further and determine whether and how plant viruses interfere with it.

Interestingly, wounding stress triggered TA of TuMV and inhibited TA of CaMV. This might indicate that the two viruses use different eliciting molecules for TA [with TuMV possibly not responding to an aphid-derived molecule but to a plant-derived wound-induced damage-associated molecular pattern (DAMP)]. Alternatively, TuMV and CaMV TA diverge after the initial recognition event. Evidence for this might come from the fact that CaMV TA stays local^7^ whereas TuMV TA might propagate through the tissue, similar to wounding that provokes tissue-wide spread of signals^31,32^. Interestingly, another TuMV protein, NIa-Pro, responds to aphid feeding not only at the feeding sites but also far away of them, indicative of signal propagation being intercepted by TuMV^33^. While propagation of TA would explain increased TuMV transmission in wounded leaves, it does not explain decreased transmission from wounded CaMV-infected leaves, where no TA propagation takes place. One would rather expect no effect of wounding on CaMV TA. The simplest explanation is that volatiles emitted from the wounded tissue^34^ deterred aphids in both cases, but the subsequent drop in transmission, due to aphid deterrence, was more than compensated by propagation of TA in TuMV-infected leaves. Clearly, more research is needed to resolve these issues.

**Figure 3:**
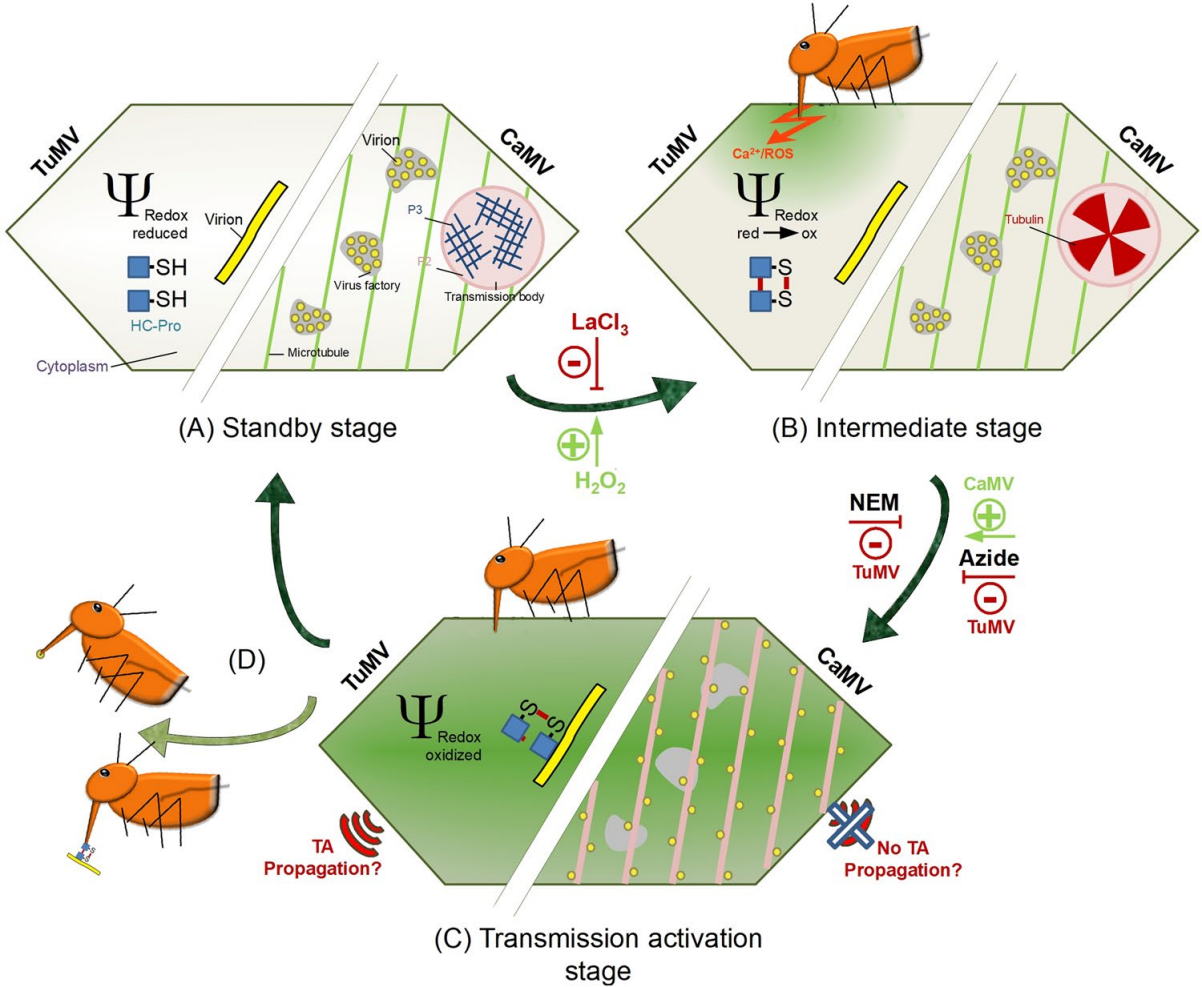
Comparison of CaMV and TuMV transmission activation. The left part of the schematized cell (not drawn to scale) presents a TuMV-infected and the right part a CaMV-infected cell (adapted from^7^). (**A**) Before aphid arrival, infected cells are in an unstressed standby stage and the cytoplasmic redox potential has normal reducing values (light green color of the cytoplasm). TuMV virions and HC-Pro are distributed evenly throughout the cytoplasm but cannot interact because HC-Pro is in its reduced conformation (-SH). CaMV virions are contained in virus factories and P2, together with P3, in transmission bodies. Thus, no or only few transmissible complexes are present. (**B**) Alighting aphids test plants by brief stylet punctures in leaf cells and inject saliva into the cytoplasm before aspiring some cell contents. Presumably, a saliva component or a DAMP binds to corresponding PRR(s) and triggers calcium and ROS signaling. Downstream events will eventually install plant defenses in a classical PTI reaction. The initial calcium waves and the accompanying ROS production change the redox potential of the cytoplasm to increasingly oxidized values (green cytoplasm). HC-Pro becomes oxidized and forms oligomers via intermolecular sulfur bridges (S-S). For CaMV, the calcium signal and the redox change induce entry of tubulin in transmission bodies. The calcium channel blocker LaCl_3_ inhibits calcium signaling, and applying H_2_O_2_ mimics ROS generation, thus explaining their effects on TA. (**C**) When the cytoplasm is maximally oxidized (dark green cytoplasm) HC-Pro oligomers bind to TuMV virions to form TuMV transmissible complexes. The inhibitory action of NEM on TuMV transmission could be by inhibiting HC-Pro oligomerization. For CaMV, the oxidizing conditions induce dissociation of the transmission bodies. Free P2 binds to microtubules and virions, dispatched from the virus factories, join P2 and form CaMV transmissible complexes. Now TuMV and CaMV infected cells are in the transmission-activated stage and vectors acquire and transmit virus efficiently. How azide inhibits TuMV and boosts CaMV transmission, is unclear. TA of TuMV but not of CaMV might propagate in the tissue. (**D**) After aphid departure, the cytoplasmic redox potential returns to reducing values, TuMV and CaMV transmissible complexes dissociate and the cells return to the standby stage.

NEM and azide treatments had different effects on TuMV and CaMV transmission. The most likely explanation is that the mechanisms of TA divert after the initial signaling event with each virus following its own pathway to TA. This is reasonable to assume because the phenology of TA is very different for the two viruses. HC-Pro of TuMV forms oxidized oligomers and subsequently interacts with virions, and CaMV helper component is released from transmission bodies to form P2-virion complexes on microtubules. As mentioned above, NEM could interfere with TuMV TA directly by inhibiting oxidation of HC-Pro, and CaMV TA might be independent of oxidation of components of the CaMV transmissible complex. The opposing effect of azide might indicate that TuMV TA requires energy, whereas energy depletion triggers TA of CaMV. It might be possible for CaMV that maintaining P2 in transmission bodies and virions in virus factories during the “standby” state (see Fig. 3) requires energy. Consequently, energy depletion might result in dissolution of transmission bodies and escape of virions from virus factories. However, as mentioned above, azide has pleiotropic effects and further research is needed to explain its action on the TA of the two viruses.

Taken together, our data that are resumed in Fig. 3 show that CaMV and TuMV TA share calcium and ROS signaling and diverge in downstream events. An obvious question is whether other viruses use TA for their transmission and if yes whether TA of these viruses also depends on calcium and ROS. Another question is whether TA of CaMV and TuMV and of potential other viruses is triggered by the same aphid and/or plant derived molecules. The answer to these questions might show the way to new strategies to control both aphids and the viruses they transmit.

## Materials and Methods

### Plants, viruses and inoculation

Turnip plants (*Brassica rapa* cv. Just Right) grown in a greenhouse at 24/15 °C day/night with a 14/10 h day/night photoperiod were used as virus hosts. Two-week-old plants were mechanically inoculated with wild-type TuMV strain UK1^35^ and with wild-type CaMV strain Cabb B-JI^36^, and used for experiments at 14 days post inoculation (dpi).

### Protoplast preparation

Protoplasts from infected turnip leaves were obtained as described^37^. Briefly, infected leaves were soaked in 2.5% diluted Domestos solution (http://www.unilever.com) for 3 min and washed with water. Then the leaves were incubated with 1% cellulase R10 and 0.05% macerozyme R10 (http://www.duchefa-biochemie.com) overnight, in the dark at 25 °C. The next day, protoplasts were filtered over one layer of Miracloth (http://www.merckmillipore.com) and washed 3 times with protoplast medium by centrifugation at 80 g in a swing-out rotor for 5 min. Protoplasts were resuspended in protoplast medium and transferred to 2 ml Eppendorf reaction tubes. Before the experiments, protoplasts were incubated at room temperature with 5 rpm agitation for 1 h to allow recovery from the protoplast preparation procedure, as reported^6,7^.

### Drug and stress treatments

Leaves on intact plants were wounded by inflicting cuts with a razor blade and immediately used for transmission tests. 10 mM H_2_O_2_ and LaCl_3_ in water was applied by spraying turnip leaves and waiting for 30 min until the leaves were dry. The negative control was spraying with water alone. For NEM treatment, 3 mM final NEM concentration was added from a 100 mM stock solution to 500 µl of protoplasts suspension and the protoplasts were incubated for 20 min before the experiments. NaN_3_ treatment was for 40 min with 0.02% final NaN_3_ concentration, added from a 10% stock solution. Protoplasts were incubated at room temperature with slow agitation (5 rpm). After treatment, protoplast viability was determined as described^38^.

### Aphid transmission assays

A non-viruliferous clonal *Myzus persicae* population was reared under controlled conditions (22/18 °C day/night with a photoperiod of 14/10 h day/night) on eggplant. The transmission tests using protoplasts and plants as virus source were performed essentially as described^7^. Briefly, apterous adult aphids were collected and starved for 1 h in metal cylinders sealed with stretched Parafilm M membranes (http://www.parafilm.com). For transmission tests using protoplasts, the cylinders were placed under a light source to attract the aphids to the membrane. Then 500 µl protoplast suspension were deposited on the membrane and covered with a cover glass and aphids were allowed an acquisition access period of 15 min. For plant-to-plant transmission tests, aphids were transferred to a leaf on an infected turnip source plant for an acquisition access period of 2 min. Then 10 aphids (protoplast experiment) or 1 aphid (plant-to-plant assays) per plant were transferred to healthy turnip test plants (cotyledon stage with the first true leaves appearing) for a 4 h inoculation period. After that, aphids were killed by application of Pirimor G aphicide (http://www.certiseurope.fr). Infected plants were identified by visual inspection for symptoms 3 weeks after inoculation. For one transmission test comprising six repetitions (five repetitions for some NEM tests), 20 plants were inoculated per repetition and per condition from aphids having had acquisition access on different cylinders (protoplast experiments) or different source plants. Each test was repeated three times, with a total of 360 plants per condition (320 plants for tests with NEM).

### Statistical analysis

Transmission rates were analyzed with generalized linear models (GLM). Quasi-binomial distributions were used in order to take overdispersion into account. For experiments involving various chemical treatments, the factors “treatment”, “date” and “manipulator” were used as explanatory variables. Statistical results are represented in the figures as box plots. The box plots were made with R 3.4.0 software.

## Supplementary information

Dataset 1

Dataset 2

Dataset 3

## Data Availability

Relevant transcriptome data and the raw data of the transmission tests and of protoplast viability are available in the Supplementary Source files.
